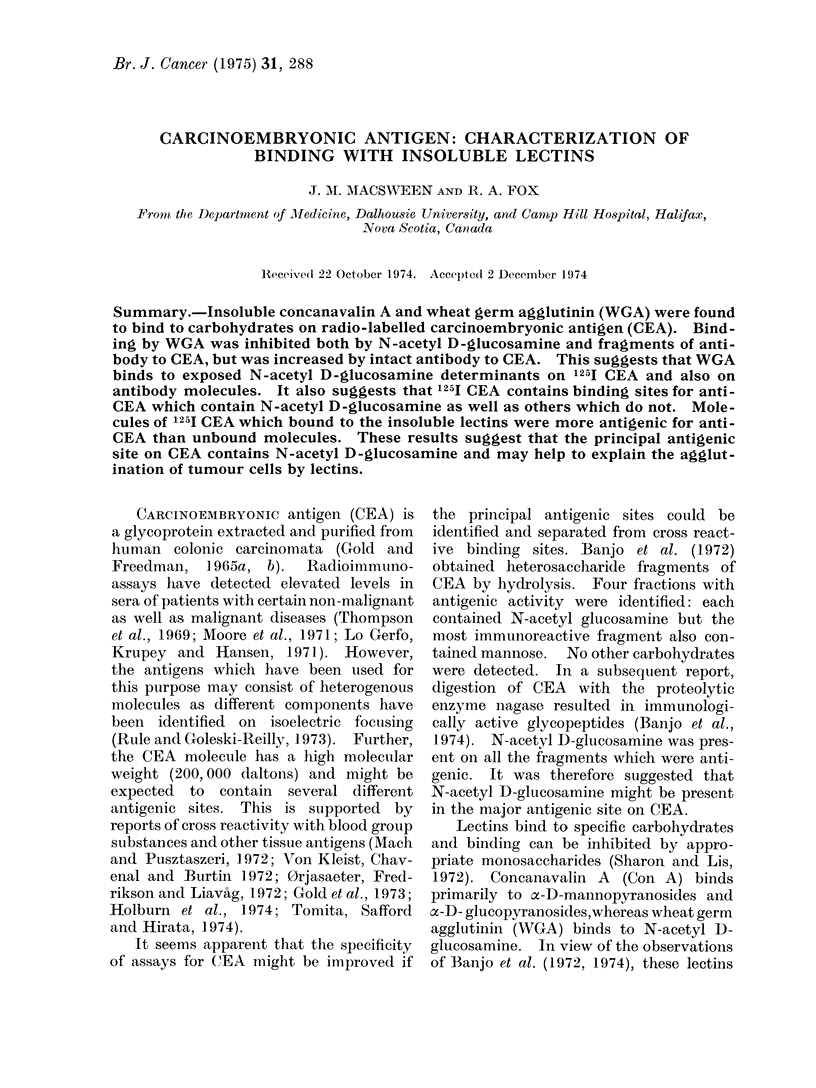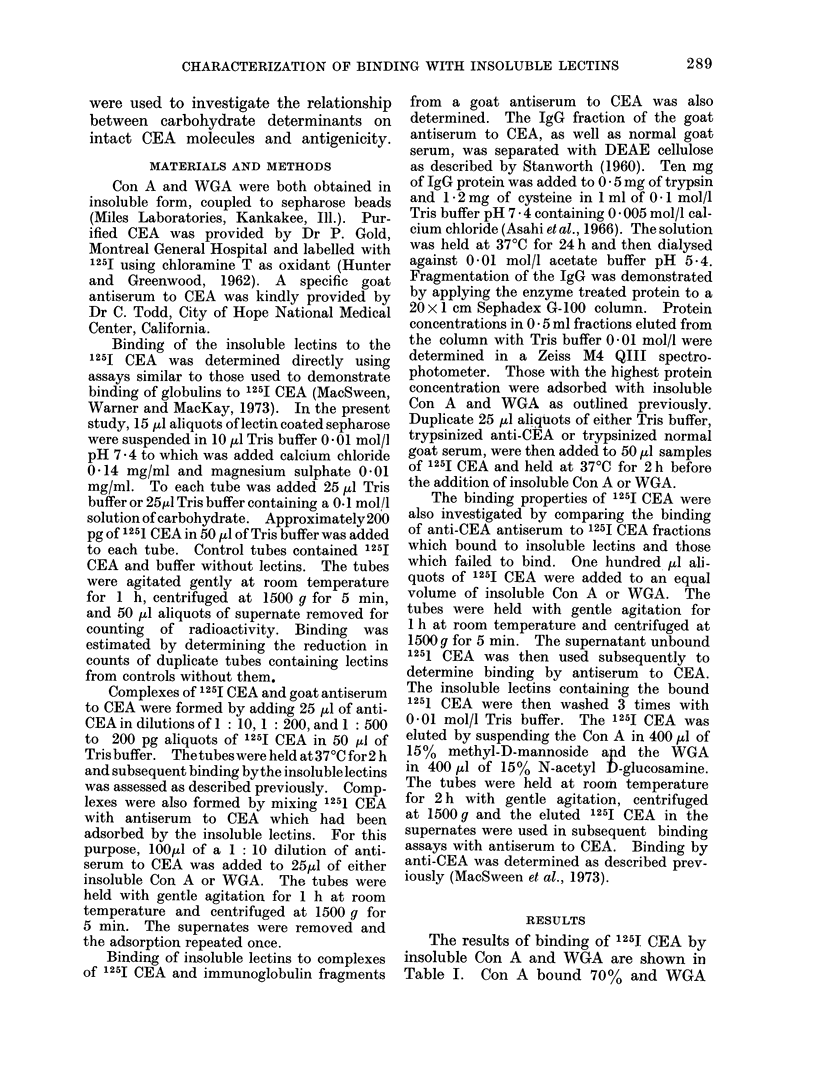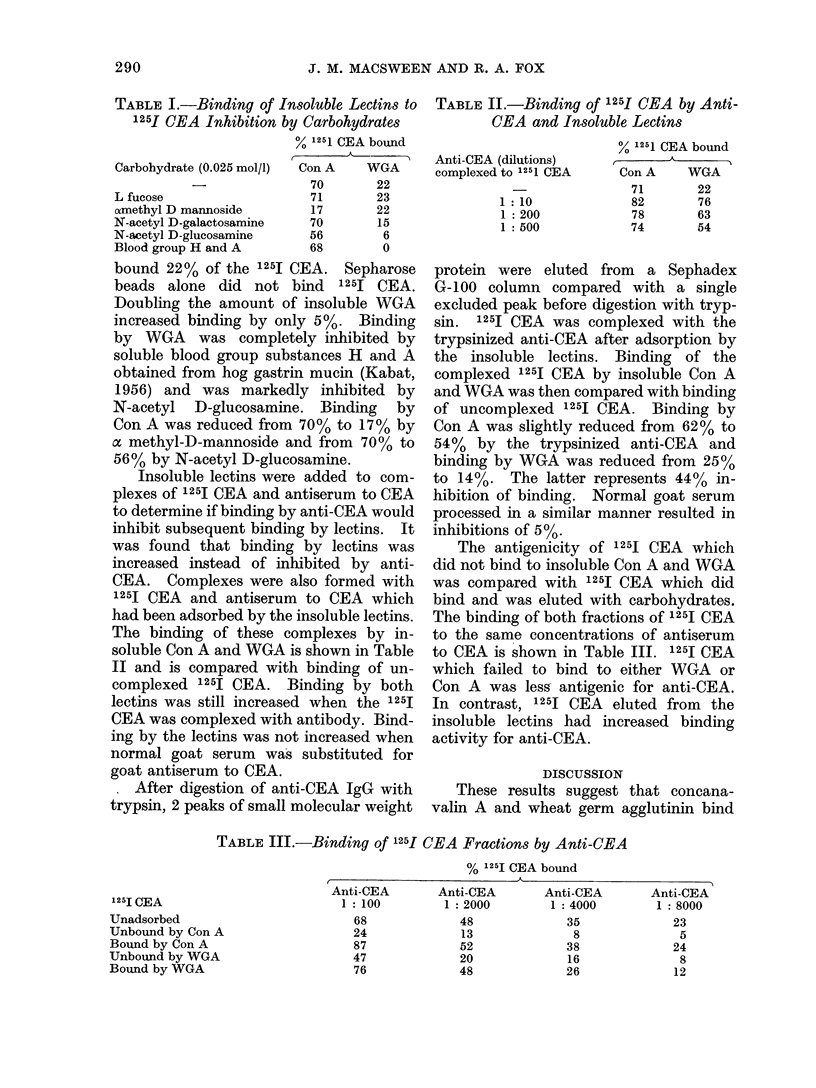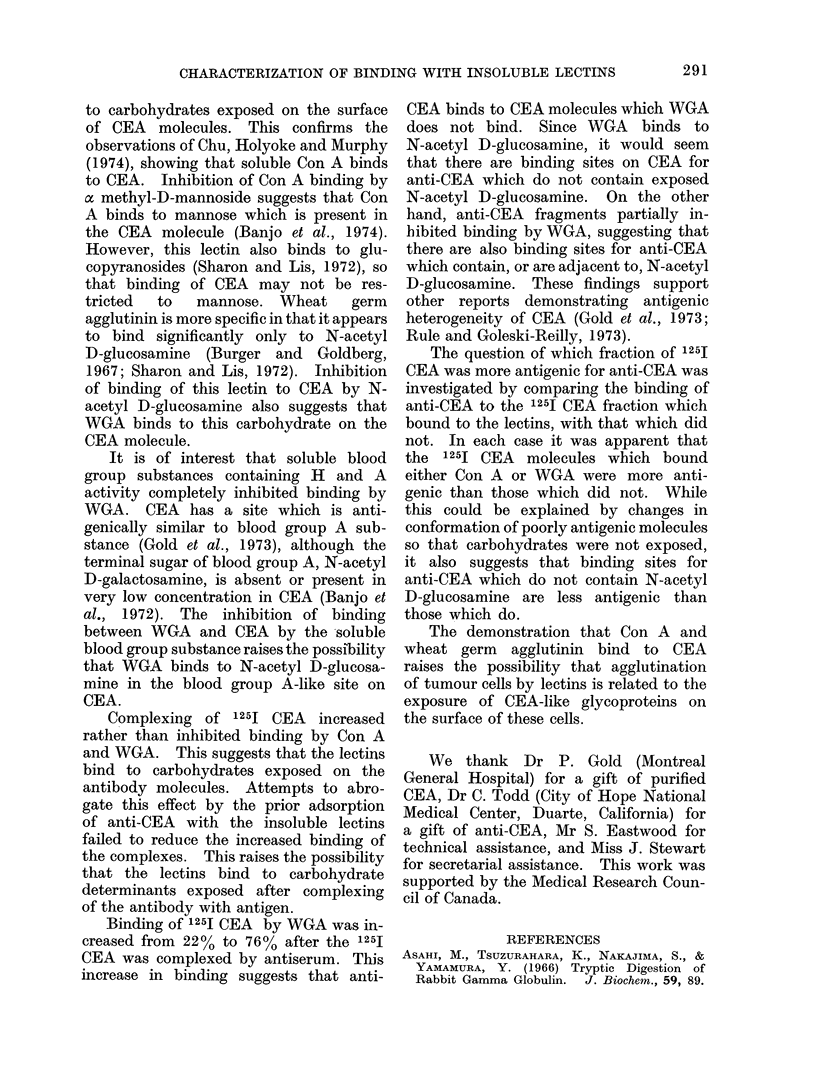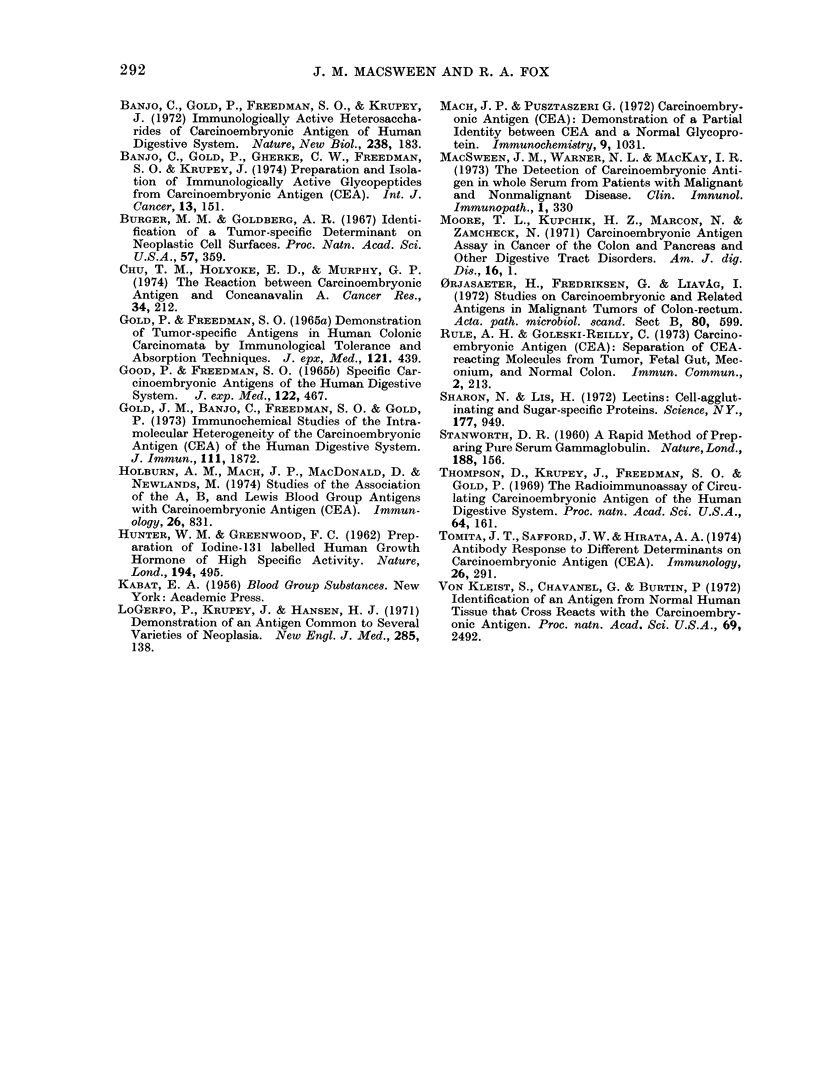# Carcinoembryonic antigen: characterization of binding with insoluble lectins.

**DOI:** 10.1038/bjc.1975.63

**Published:** 1975-03

**Authors:** J. M. Macsween, R. A. Fox

## Abstract

Insoluble conanavalin A and wheat germ agglutinin (WGA) were found to bind to carbohydrates on radio-labelled carcinoembryonic antigen (CEA). Binding by WGA was inhibited both by N-acetyl D-glucosamine and fragments of antibody to CEA, but was increased by intact antibody to CEA. This suggests that WGA binds to exposed N-acetyl D-glucosamine determinants on 125I CEA and also on antibody molecules. It also suggests that 125I CEA contains binding sites for anti-CEA which contain N-acetyl D-glucosamine as well as others which do not. Molecules of 125I CEA which bound to the insoluble lectins were more antigenic for anti-CEA than unbound molecules. These results suggest that the principal antigenic site on CEA contains N-acetyl D-glucosamine and may help to explain the agglutination of tumour cells by lectins.


*Br. J. Cancer* (1975) **31**, 288

### CARCINOEMBRYONIC ANTIGEN: CHARACTERIZATION OF BINDING WITH INSOLUBLE LECTINS

J. M. MACSWEEN AND R. A. FOX

*From the Department of Medicine, Dalhousie University, and Camp Hill Hospital, Halifax, Nova Scotia, Canada*

Received 22 October 1974. Accepted 2 December 1974

**Summary.—Insoluble concanavalin A and wheat germ agglutinin (WGA) were found to bind to carbohydrates on radio-labelled carcinoembryonic antigen (CEA). Binding by WGA was inhibited both by N-acetyl D-glucosamine and fragments of antibody to CEA, but was increased by intact antibody to CEA. This suggests that WGA binds to exposed N-acetyl D-glucosamine determinants on $^{125}$I CEA and also on antibody molecules. It also suggests that $^{125}$I CEA contains binding sites for anti-CEA which contain N-acetyl D-glucosamine as well as others which do not. Molecules of $^{125}$I CEA which bound to the insoluble lectins were more antigenic for anti-CEA than unbound molecules. These results suggest that the principal antigenic site on CEA contains N-acetyl D-glucosamine and may help to explain the agglutination of tumour cells by lectins.**

CARCINOEMBRYONIC antigen (CEA) is a glycoprotein extracted and purified from human colonic carcinomata (Gold and Freedman, 1965*a*, *b*). Radioimmunoassays have detected elevated levels in sera of patients with certain non-malignant as well as malignant diseases (Thompson *et al.*, 1969; Moore *et al.*, 1971; Lo Gerfo, Krupey and Hansen, 1971). However, the antigens which have been used for this purpose may consist of heterogenous molecules as different components have been identified on isoelectric focusing (Rule and Goleski-Reilly, 1973). Further, the CEA molecule has a high molecular weight (200,000 daltons) and might be expected to contain several different antigenic sites. This is supported by reports of cross reactivity with blood group substances and other tissue antigens (Mach and Pusztaszeri, 1972; Von Kleist, Chavenal and Burtin 1972; Ørjasaeter, Fredrikson and Liavåg, 1972; Gold *et al.*, 1973; Holburn *et al.*, 1974; Tomita, Safford and Hirata, 1974).

It seems apparent that the specificity of assays for CEA might be improved if the principal antigenic sites could be identified and separated from cross reactive binding sites. Banjo *et al.* (1972) obtained heterosaccharide fragments of CEA by hydrolysis. Four fractions with antigenic activity were identified: each contained N-acetyl glucosamine but the most immunoreactive fragment also contained mannose. No other carbohydrates were detected. In a subsequent report, digestion of CEA with the proteolytic enzyme nagase resulted in immunologically active glycopeptides (Banjo *et al.*, 1974). N-acetyl D-glucosamine was present on all the fragments which were antigenic. It was therefore suggested that N-acetyl D-glucosamine might be present in the major antigenic site on CEA.

Lectins bind to specific carbohydrates and binding can be inhibited by appropriate monosaccharides (Sharon and Lis, 1972). Concanavalin A (Con A) binds primarily to $\alpha$-D-mannopyranosides and $\alpha$-D-glucopyranosides, whereas wheat germ agglutinin (WGA) binds to N-acetyl D-glucosamine. In view of the observations of Banjo *et al.* (1972, 1974), these lectins were used to investigate the relationship between carbohydrate determinants on intact CEA molecules and antigenicity.

### MATERIALS AND METHODS

Con A and WGA were both obtained in insoluble form, coupled to sepharose beads (Miles Laboratories, Kankakee, Ill.). Purified CEA was provided by Dr P. Gold, Montreal General Hospital and labelled with $^{125}$I using chloramine T as oxidant (Hunter and Greenwood, 1962). A specific goat antiserum to CEA was kindly provided by Dr C. Todd, City of Hope National Medical Center, California.

Binding of the insoluble lectins to the $^{125}$I CEA was determined directly using assays similar to those used to demonstrate binding of globulins to $^{125}$I CEA (MacSween, Warner and MacKay, 1973). In the present study, 15 $\mu$l aliquots of lectin coated sepharose were suspended in 10 $\mu$l Tris buffer 0·01 mol/l pH 7·4 to which was added calcium chloride 0·14 mg/ml and magnesium sulphate 0·01 mg/ml. To each tube was added 25 $\mu$l Tris buffer or 25$\mu$l Tris buffer containing a 0·1 mol/l solution of carbohydrate. Approximately 200 pg of $^{125}$I CEA in 50 $\mu$l of Tris buffer was added to each tube. Control tubes contained $^{125}$I CEA and buffer without lectins. The tubes were agitated gently at room temperature for 1 h, centrifuged at 1500 *g* for 5 min, and 50 $\mu$l aliquots of supernate removed for counting of radioactivity. Binding was estimated by determining the reduction in counts of duplicate tubes containing lectins from controls without them.

Complexes of $^{125}$I CEA and goat antiserum to CEA were formed by adding 25 $\mu$l of anti-CEA in dilutions of 1 : 10, 1 : 200, and 1 : 500 to 200 pg aliquots of $^{125}$I CEA in 50 $\mu$l of Tris buffer. The tubes were held at 37°C for 2 h and subsequent binding by the insoluble lectins was assessed as described previously. Complexes were also formed by mixing $^{125}$I CEA with antiserum to CEA which had been adsorbed by the insoluble lectins. For this purpose, 100$\mu$l of a 1 : 10 dilution of antiserum to CEA was added to 25$\mu$l of either insoluble Con A or WGA. The tubes were held with gentle agitation for 1 h at room temperature and centrifuged at 1500 *g* for 5 min. The supernates were removed and the adsorption repeated once.

Binding of insoluble lectins to complexes of $^{125}$I CEA and immunoglobulin fragments from a goat antiserum to CEA was also determined. The IgG fraction of the goat antiserum to CEA, as well as normal goat serum, was separated with DEAE cellulose as described by Stanworth (1960). Ten mg of IgG protein was added to 0·5 mg of trypsin and 1·2 mg of cysteine in 1 ml of 0·1 mol/l Tris buffer pH 7·4 containing 0·005 mol/l calcium chloride (Asahi *et al.*, 1966). The solution was held at 37°C for 24 h and then dialysed against 0·01 mol/l acetate buffer pH 5·4. Fragmentation of the IgG was demonstrated by applying the enzyme treated protein to a 20 × 1 cm Sephadex G-100 column. Protein concentrations in 0·5 ml fractions eluted from the column with Tris buffer 0·01 mol/l were determined in a Zeiss M4 QIII spectrophotometer. Those with the highest protein concentration were adsorbed with insoluble Con A and WGA as outlined previously. Duplicate 25 $\mu$l aliquots of either Tris buffer, trypsinized anti-CEA or trypsinized normal goat serum, were then added to 50 $\mu$l samples of $^{125}$I CEA and held at 37°C for 2 h before the addition of insoluble Con A or WGA.

The binding properties of $^{125}$I CEA were also investigated by comparing the binding of anti-CEA antiserum to $^{125}$I CEA fractions which bound to insoluble lectins and those which failed to bind. One hundred $\mu$l aliquots of $^{125}$I CEA were added to an equal volume of insoluble Con A or WGA. The tubes were held with gentle agitation for 1 h at room temperature and centrifuged at 1500 *g* for 5 min. The supernatant unbound $^{125}$I CEA was then used subsequently to determine binding by antiserum to CEA. The insoluble lectins containing the bound $^{125}$I CEA were then washed 3 times with 0·01 mol/l Tris buffer. The $^{125}$I CEA was eluted by suspending the Con A in 400 $\mu$l of 15% methyl-D-mannoside and the WGA in 400 $\mu$l of 15% N-acetyl D-glucosamine. The tubes were held at room temperature for 2 h with gentle agitation, centrifuged at 1500 *g* and the eluted $^{125}$I CEA in the supernates were used in subsequent binding assays with antiserum to CEA. Binding by anti-CEA was determined as described previously (MacSween *et al.*, 1973).

### RESULTS

The results of binding of $^{125}$I CEA by insoluble Con A and WGA are shown in Table I. Con A bound 70% and WGA

TABLE I.—*Binding of Insoluble Lectins to $^{125}I$ CEA Inhibition by Carbohydrates*

| Carbohydrate (0.025 mol/l) | % $^{125}$I CEA bound Con A | WGA |
|---|---|---|
| — | 70 | 22 |
| L fucose | 71 | 23 |
| αmethyl D mannoside | 17 | 22 |
| N-acetyl D-galactosamine | 70 | 15 |
| N-acetyl D-glucosamine | 56 | 6 |
| Blood group H and A | 68 | 0 |

TABLE II.—*Binding of $^{125}I$ CEA by Anti-CEA and Insoluble Lectins*

| Anti-CEA (dilutions) complexed to $^{125}$I CEA | % $^{125}$I CEA bound Con A | WGA |
|---|---|---|
| — | 71 | 22 |
| 1 : 10 | 82 | 76 |
| 1 : 200 | 78 | 63 |
| 1 : 500 | 74 | 54 |

bound 22% of the $^{125}$I CEA. Sepharose beads alone did not bind $^{125}$I CEA. Doubling the amount of insoluble WGA increased binding by only 5%. Binding by WGA was completely inhibited by soluble blood group substances H and A obtained from hog gastrin mucin (Kabat, 1956) and was markedly inhibited by N-acetyl D-glucosamine. Binding by Con A was reduced from 70% to 17% by α methyl-D-mannoside and from 70% to 56% by N-acetyl D-glucosamine.

Insoluble lectins were added to complexes of $^{125}$I CEA and antiserum to CEA to determine if binding by anti-CEA would inhibit subsequent binding by lectins. It was found that binding by lectins was increased instead of inhibited by anti-CEA. Complexes were also formed with $^{125}$I CEA and antiserum to CEA which had been adsorbed by the insoluble lectins. The binding of these complexes by insoluble Con A and WGA is shown in Table II and is compared with binding of uncomplexed $^{125}$I CEA. Binding by both lectins was still increased when the $^{125}$I CEA was complexed with antibody. Binding by the lectins was not increased when normal goat serum was substituted for goat antiserum to CEA.

After digestion of anti-CEA IgG with trypsin, 2 peaks of small molecular weight protein were eluted from a Sephadex G-100 column compared with a single excluded peak before digestion with trypsin. $^{125}$I CEA was complexed with the trypsinized anti-CEA after adsorption by the insoluble lectins. Binding of the complexed $^{125}$I CEA by insoluble Con A and WGA was then compared with binding of uncomplexed $^{125}$I CEA. Binding by Con A was slightly reduced from 62% to 54% by the trypsinized anti-CEA and binding by WGA was reduced from 25% to 14%. The latter represents 44% inhibition of binding. Normal goat serum processed in a similar manner resulted in inhibitions of 5%.

The antigenicity of $^{125}$I CEA which did not bind to insoluble Con A and WGA was compared with $^{125}$I CEA which did bind and was eluted with carbohydrates. The binding of both fractions of $^{125}$I CEA to the same concentrations of antiserum to CEA is shown in Table III. $^{125}$I CEA which failed to bind to either WGA or Con A was less antigenic for anti-CEA. In contrast, $^{125}$I CEA eluted from the insoluble lectins had increased binding activity for anti-CEA.

### DISCUSSION

These results suggest that concanavalin A and wheat germ agglutinin bind

TABLE III.—*Binding of $^{125}I$ CEA Fractions by Anti-CEA*

| $^{125}$I CEA | % $^{125}$I CEA bound Anti-CEA 1 : 100 | Anti-CEA 1 : 2000 | Anti-CEA 1 : 4000 | Anti-CEA 1 : 8000 |
|---|---|---|---|---|
| Unadsorbed | 68 | 48 | 35 | 23 |
| Unbound by Con A | 24 | 13 | 8 | 5 |
| Bound by Con A | 87 | 52 | 38 | 24 |
| Unbound by WGA | 47 | 20 | 16 | 8 |
| Bound by WGA | 76 | 48 | 26 | 12 |

to carbohydrates exposed on the surface of CEA molecules. This confirms the observations of Chu, Holyoke and Murphy (1974), showing that soluble Con A binds to CEA. Inhibition of Con A binding by $\alpha$ methyl-D-mannoside suggests that Con A binds to mannose which is present in the CEA molecule (Banjo *et al.*, 1974). However, this lectin also binds to glucopyranosides (Sharon and Lis, 1972), so that binding of CEA may not be restricted to mannose. Wheat germ agglutinin is more specific in that it appears to bind significantly only to N-acetyl D-glucosamine (Burger and Goldberg, 1967; Sharon and Lis, 1972). Inhibition of binding of this lectin to CEA by N-acetyl D-glucosamine also suggests that WGA binds to this carbohydrate on the CEA molecule.

It is of interest that soluble blood group substances containing H and A activity completely inhibited binding by WGA. CEA has a site which is antigenically similar to blood group A substance (Gold *et al.*, 1973), although the terminal sugar of blood group A, N-acetyl D-galactosamine, is absent or present in very low concentration in CEA (Banjo *et al.*, 1972). The inhibition of binding between WGA and CEA by the soluble blood group substance raises the possibility that WGA binds to N-acetyl D-glucosamine in the blood group A-like site on CEA.

Complexing of $^{125}$I CEA increased rather than inhibited binding by Con A and WGA. This suggests that the lectins bind to carbohydrates exposed on the antibody molecules. Attempts to abrogate this effect by the prior adsorption of anti-CEA with the insoluble lectins failed to reduce the increased binding of the complexes. This raises the possibility that the lectins bind to carbohydrate determinants exposed after complexing of the antibody with antigen.

Binding of $^{125}$I CEA by WGA was increased from 22% to 76% after the $^{125}$I CEA was complexed by antiserum. This increase in binding suggests that anti-CEA binds to CEA molecules which WGA does not bind. Since WGA binds to N-acetyl D-glucosamine, it would seem that there are binding sites on CEA for anti-CEA which do not contain exposed N-acetyl D-glucosamine. On the other hand, anti-CEA fragments partially inhibited binding by WGA, suggesting that there are also binding sites for anti-CEA which contain, or are adjacent to, N-acetyl D-glucosamine. These findings support other reports demonstrating antigenic heterogeneity of CEA (Gold *et al.*, 1973; Rule and Goleski-Reilly, 1973).

The question of which fraction of $^{125}$I CEA was more antigenic for anti-CEA was investigated by comparing the binding of anti-CEA to the $^{125}$I CEA fraction which bound to the lectins, with that which did not. In each case it was apparent that the $^{125}$I CEA molecules which bound either Con A or WGA were more antigenic than those which did not. While this could be explained by changes in conformation of poorly antigenic molecules so that carbohydrates were not exposed, it also suggests that binding sites for anti-CEA which do not contain N-acetyl D-glucosamine are less antigenic than those which do.

The demonstration that Con A and wheat germ agglutinin bind to CEA raises the possibility that agglutination of tumour cells by lectins is related to the exposure of CEA-like glycoproteins on the surface of these cells.

We thank Dr P. Gold (Montreal General Hospital) for a gift of purified CEA, Dr C. Todd (City of Hope National Medical Center, Duarte, California) for a gift of anti-CEA, Mr S. Eastwood for technical assistance, and Miss J. Stewart for secretarial assistance. This work was supported by the Medical Research Council of Canada.

### REFERENCES

Asahi, M., Tsuzurahara, K., Nakajima, S., & Yamamura, Y. (1966) Tryptic Digestion of Rabbit Gamma Globulin. *J. Biochem.*, **59**, 89.

BANJO, C., GOLD, P., FREEDMAN, S. O., & KRUPEY, J. (1972) Immunologically Active Heterosaccharides of Carcinoembryonic Antigen of Human Digestive System. *Nature, New Biol.*, **238**, 183.

BANJO, C., GOLD, P., GHERKE, C. W., FREEDMAN, S. O. & KRUPEY, J. (1974) Preparation and Isolation of Immunologically Active Glycopeptides from Carcinoembryonic Antigen (CEA). *Int. J. Cancer*, **13**, 151.

BURGER, M. M. & GOLDBERG, A. R. (1967) Identification of a Tumor-specific Determinant on Neoplastic Cell Surfaces. *Proc. Natn. Acad. Sci. U.S.A.*, **57**, 359.

CHU, T. M., HOLYOKE, E. D., & MURPHY, G. P. (1974) The Reaction between Carcinoembryonic Antigen and Concanavalin A. *Cancer Res.*, **34**, 212.

GOLD, P. & FREEDMAN, S. O. (1965*a*) Demonstration of Tumor-specific Antigens in Human Colonic Carcinomata by Immunological Tolerance and Absorption Techniques. *J. epx, Med.*, **121**. 439.

GOOD, P. & FREEDMAN, S. O. (1965*b*) Specific Carcinoembryonic Antigens of the Human Digestive System. *J. exp. Med.*, **122**, 467.

GOLD, J. M., BANJO, C., FREEDMAN, S. O. & GOLD, P. (1973) Immunochemical Studies of the Intramolecular Heterogeneity of the Carcinoembryonic Antigen (CEA) of the Human Digestive System. *J. Immun.*, **111**, 1872.

HOLBURN, A. M., MACH, J. P., MACDONALD, D. & NEWLANDS, M. (1974) Studies of the Association of the A, B, and Lewis Blood Group Antigens with Carcinoembryonic Antigen (CEA). *Immunology*, **26**, 831.

HUNTER, W. M. & GREENWOOD, F. C. (1962) Preparation of Iodine-131 labelled Human Growth Hormone of High Specific Activity. *Nature, Lond.*, **194**, 495.

KABAT, E. A. (1956) *Blood Group Substances*. New York: Academic Press.

LOGERFO, P., KRUPEY, J. & HANSEN, H. J. (1971) Demonstration of an Antigen Common to Several Varieties of Neoplasia. *New Engl. J. Med.*, **285**, 138.

MACH, J. P. & PUSZTASZERI G. (1972) Carcinoembryonic Antigen (CEA): Demonstration of a Partial Identity between CEA and a Normal Glycoprotein. *Immunochemistry*, **9**, 1031.

MACSWEEN, J. M., WARNER, N. L. & MACKAY, I. R. (1973) The Detection of Carcinoembryonic Antigen in whole Serum from Patients with Malignant and Nonmalignant Disease. *Clin. Immunol. Immunopath.*, **1**, 330

MOORE, T. L., KUPCHIK, H. Z., MARCON, N. & ZAMCHECK, N. (1971) Carcinoembryonic Antigen Assay in Cancer of the Colon and Pancreas and Other Digestive Tract Disorders. *Am. J. dig. Dis.*, **16**, 1.

ØRJASAETER, H., FREDRIKSEN, G. & LIAVÅG, I. (1972) Studies on Carcinoembryonic and Related Antigens in Malignant Tumors of Colon-rectum. *Acta. path. microbiol. scand.* Sect B, **80**, 599.

RULE, A. H. & GOLESKI-REILLY, C. (1973) Carcinoembryonic Antigen (CEA): Separation of CEA-reacting Molecules from Tumor, Fetal Gut, Meconium, and Normal Colon. *Immun. Commun.*, **2**, 213.

SHARON, N. & LIS, H. (1972) Lectins: Cell-agglutinating and Sugar-specific Proteins. *Science, NY.*, **177**, 949.

STANWORTH, D. R. (1960) A Rapid Method of Preparing Pure Serum Gammaglobulin. *Nature, Lond.*, **188**, 156.

THOMPSON, D., KRUPEY, J., FREEDMAN, S. O. & GOLD, P. (1969) The Radioimmunoassay of Circulating Carcinoembryonic Antigen of the Human Digestive System. *Proc. natn. Acad. Sci. U.S.A.*, **64**, 161.

TOMITA, J. T., SAFFORD, J. W. & HIRATA, A. A. (1974) Antibody Response to Different Determinants on Carcinoembryonic Antigen (CEA). *Immunology*, **26**, 291.

VON KLEIST, S., CHAVANEL, G. & BURTIN, P (1972) Identification of an Antigen from Normal Human Tissue that Cross Reacts with the Carcinoembryonic Antigen. *Proc. natn. Acad. Sci. U.S.A.*, **69**, 2492.